# Developing a Selection-aided Model to Screen Cirrhotic Intrahepatic Cholangiocarcinoma for Hepatectomy

**DOI:** 10.7150/jca.46587

**Published:** 2020-07-25

**Authors:** Qinjunjie Chen, Fengwei Li, Yuzhen Gao, Hui Xue, Zheng Li, Qifei Zou, Yong Xia, Kui Wang, Feng Shen

**Affiliations:** 1 Department of Hepatic Surgery IV, the Eastern Hepatobiliary Surgery Hospital, Naval Medical University (Second Military Medical University), Shanghai, China.; 2 Department of Hepatic Surgery II, the Eastern Hepatobiliary Surgery Hospital, Naval Medical University (Second Military Medical University), Shanghai, China.; 3 Department of Molecular Diagnosis, Clinical Medical College, Yangzhou University, Jiangsu, China.

**Keywords:** intrahepatic cholangiocarcinoma, cirrhosis, liver resection, prognosis

## Abstract

**Background:** This study aimed to establish a model predicting the prognosis of intrahepatic cholangiocarcinoma (ICC) patients with cirrhosis before liver resection (LR).

**Methods:** An Eastern Hepatobiliary Surgery Hospital (EHBH) model using the preoperative factors was established in a training cohort (305 patients from 2006 to 2011) and validated in an internal validation cohort (113 patients from 2012 to 2014). Predictive performance and discrimination were evaluated and compared with other staging systems.

**Results:** The EHBH model containing preoperative factors of carbohydrate antigen 19-9 (CA19-9), radiological tumor diameter, tumor number, and satellite nodules outperformed other staging systems in predicting the prognosis of ICC. A contour plot of 3-year survival probability and a nomogram to form two differentiated groups of patients (high-risk group and low-risk group) were constructed based on the EHBH model to help surgeons predicting the overall survival (OS) before LR. Patients from the high-risk group (>86.56 points) in the training cohort had worse OS rates compared with those from the low-risk group (≤86.56 points). The one-, three-, and five-year OS rates were 50.4%, 29.0%, and 21.0% for the high-risk group and 68.2%, 45.5%, and 39.7% for the low-risk group, respectively (*P*<0.001). The same results were obtained in the internal validation patients.

**Conclusion:** The contour plot is an easy-to-use tool to individually show the 3-year prognosis of ICC patients with different preoperative CA19-9 values and radiological characteristics before surgery. The EHBH model was suitable for selecting cirrhotic patients for LR to acquire a better survival.

## Introduction

Intrahepatic cholangiocarcinoma (ICC) is a highly aggressive liver malignancy disease. The incidence of ICC, which is only second to hepatocellular carcinoma (HCC), has increased globally over the past decades 1, 2. ICC frequently combined with large and/or multiple tumors because of late diagnoses which are characterized as negative prognostic factors 3, 4. At present, liver resection (LR) is the only established treatment to achieve the possible long-term survival of ICC patients 5. However, even if the patient underwent radical surgery, early postoperative recurrence of ICC usually led to a poor long-term survival outcome.

Presently, the best efficient management for patients with multiple tumors remains controversial, especially for those with cirrhosis 6, and tumor multinodularity is attracting considerable attention because of its prognostic value in ICC 6-12. If we could select proper patients for LR to help them acquire a good postoperative survival, some patients with poor postoperative prognosis would avoid meaningless surgery.

To that end, our study presented a simple model (Eastern Hepatobiliary Surgery Hospital model, EHBH model) based on preoperative radiological examinations and serological index, which was further validated using the internal validation cohort to accurately predict the long-term survival in cirrhotic patients before surgery. The proposed model can serve as a supplement to international staging systems in the selection of subgroups of ICC patients for surgical treatments.

## Materials and Methods

### Study population

A total of 305 consecutive patients who underwent LR for histopathologically confirmed ICC between January 2006 and December 2011 at EHBH was used as training cohort and 113 patients from the same center (between January 2012 and December 2014) was used as an internal validation cohort. This study was approved by the Institutional Ethics Committee of EHBH. Informed consent was obtained from all patients before surgery in this study.

### Inclusion and exclusion criteria

The inclusion criteria for ICC patients were expressed as follows: (1) patients with good performance (ECOG 0-2 score) without obvious heart, lung, kidney, and other important organ diseases, (2) Child-Pugh grade A or B7 liver function without refractory ascites, (3) local tumor or multiple tumors in adjacent segments of the liver without any evidence of distant tumor metastasis. The segments or lobes can be removed or local excision can be performed with future liver remnant ≥ 50% using the measurement of preoperative computed tomography/magnetic resonance imaging (CT/MRI). (4) Mild to moderate cirrhosis, and esophagus and gastric varices with moderate to severe without bleeding tendency, namely, red color sign. No history of variceal bleeding, (5) platelet count ≥ 75 × 10^9^/L. The exclusion criteria were expressed as follows: patients with (1) Child-Pugh class C liver function, (2) incomplete pre- or post-operative data.

The results of the training cohort were validated in one internal validation cohort using the same inclusion/exclusion criteria.

### LR and definitions

Routine preoperative laboratory and imaging examinations were conducted, as previously reported 3. All operations were conducted for the complete removal of macroscopic tumors with adequate resection margins. The detailed surgical procedures were similar to our previously reported procedures 3. Intraoperative ultrasound was routinely used. The range of hepatectomy was determined by the operating surgeons based on tumor stage, distribution, liver function, cirrhosis, and estimated volume of the future liver remnant. Dissection of regional lymph nodes was not routinely performed with small ICC who had no clinical evidence of nodal metastasis preoperatively or with patients of postoperatively pathological diagnosed ICC. Direct invasion of adjacent tissues and newly found intrahepatic nodules identified intraoperatively were removed whenever possible. Histopathological examination of surgical specimens was routinely conducted. The histopathological diagnosis of ICC was based on the WHO classification 7.

The presence of preoperative CSPH was based on the definition of standard surrogate criteria proposed by the Barcelona Clinic Liver Cancer (BCLC) classification, which was defined as the presence of esophagogastric varices or splenomegaly (diameter >12 cm) with a platelet count < 100 × 10^9^/L 13.

Child-Pugh score was calculated as previously proposed 14. Tumor stages or scores were categorized based on the revised liver cancer study group of Japan (LCSGJ) staging systems 15, the 8th edition of the American Joint Committee on Cancer (AJCC) 16, the Japanese Society of Hepato-Biliary-Pancreatic Surgery (JSHBPS) staging system 17, the Fudan scoring system 18, and the Zhou scoring system for ICC 19.

### Follow-up and endpoints

Patients were followed-up once every 2 months within the first 2 years after surgery and once every 3-6 months. At each visit, liver and renal functions, alpha fetoprotein (AFP), carbohydrate antigen 19-9 (CA19-9), and carcinoembryonic antigen (CEA) were evaluated, and an abdominal ultrasound was performed. Contrast-enhanced CT scan or MRI was conducted once every 4-6 months or earlier if clinically indicated. The endpoints were overall survival (OS), which was calculated from the date of surgery to the date of patient death or last follow-up, and recurrence-free survival (RFS) was defined from the date of surgery to the date when HCC recurrence was first diagnosed.

### Statistical analysis

Continuous variables were reported as means with standard deviations or medians with interquartile range. They were compared using student's *t*-test or Mann-Whitney *U* test. Categorical data, presented as frequencies (%), were compared using chi-square or Fisher's exact tests. To establish the EHBH model and the nomogram, we selected the outcomes of multivariable Cox regression analyses of OS with a stepwise method by using the “rms” package of R, version 3.4 (http://www.r-project.org/). Then, a restricted cubic spline (RCS) was used to find the nonlinear relationship between the variables and OS. Moreover, predicted probability and risk contour plots were used to display the predicted risk of individual patients.

We used several methods to validate the effectiveness of the EHBH model. First, the prediction accuracies of the EHBH model and other clinical other staging systems were calculated based on Harrell's c-statistics 20. Then, the nomogram scores of each patient were calculated and then the best cut-off score of the nomogram was detected by the receiver operating characteristic curve (ROC) to divide patients into the high-risk group and low-risk group, and Kaplan-Meier curves of OS or RFS were calculated in different cohorts and groups. Decision curve analysis (DCA) of the EHBH model was made to validate its performance in the training cohort. Time-dependent areas under the receiver operating characteristic curve (AUC) of each point in different cohorts were measured from 10 months to 80 months, reflecting the performance in predicting OS at various time points. All the reported p values were the results of two-sided tests. A significance level of 0.05 was applied. All statistical analyses were carried out in the R program, version 3.4 (http://www.r-project.org/) (Vienna, Austria, version 3.4.4), and SPSS (IBM, version 23, USA).

## Results

### Patient characteristics

**Figure 1:**
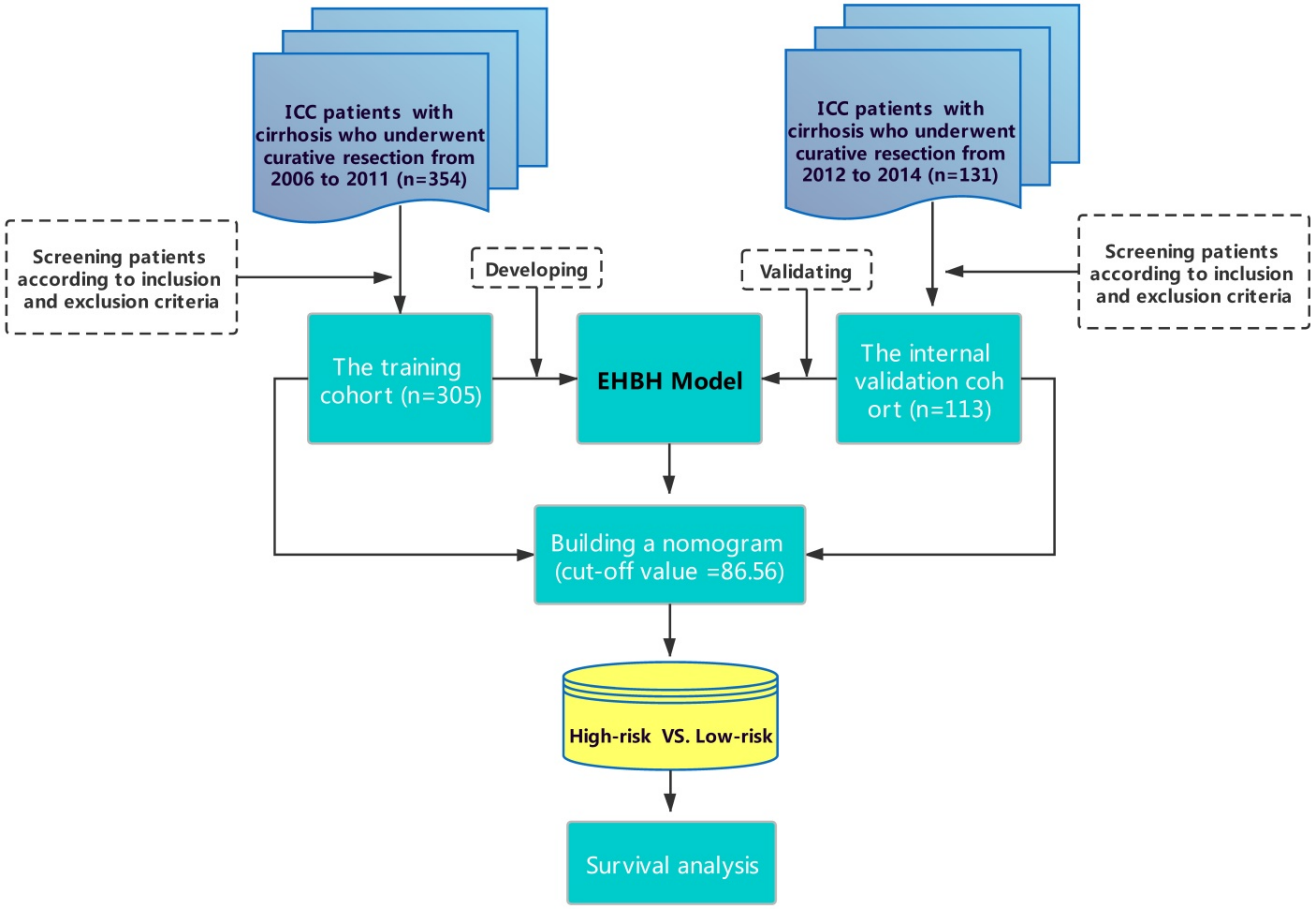
Flowchart of this study.

As shown in Figure 1, 305 patients with cirrhosis at EHBH met the inclusion criteria, which were enrolled in the training cohort. The internal validation cohort of 113 ICC patients was obtained from an independent cohort of consecutive patients with cirrhosis in EHBH. Table 1 summarizes the baseline clinicopathological features of the patients between the two cohorts. Significant differences were only observed in the percentage of satellite nodules among the two cohorts. Kaplan-curves of OS and RFS between the two cohorts were shown in Figures S1A, S1B.

### Univariable and multivariable Cox analyses

**Figure 2:**
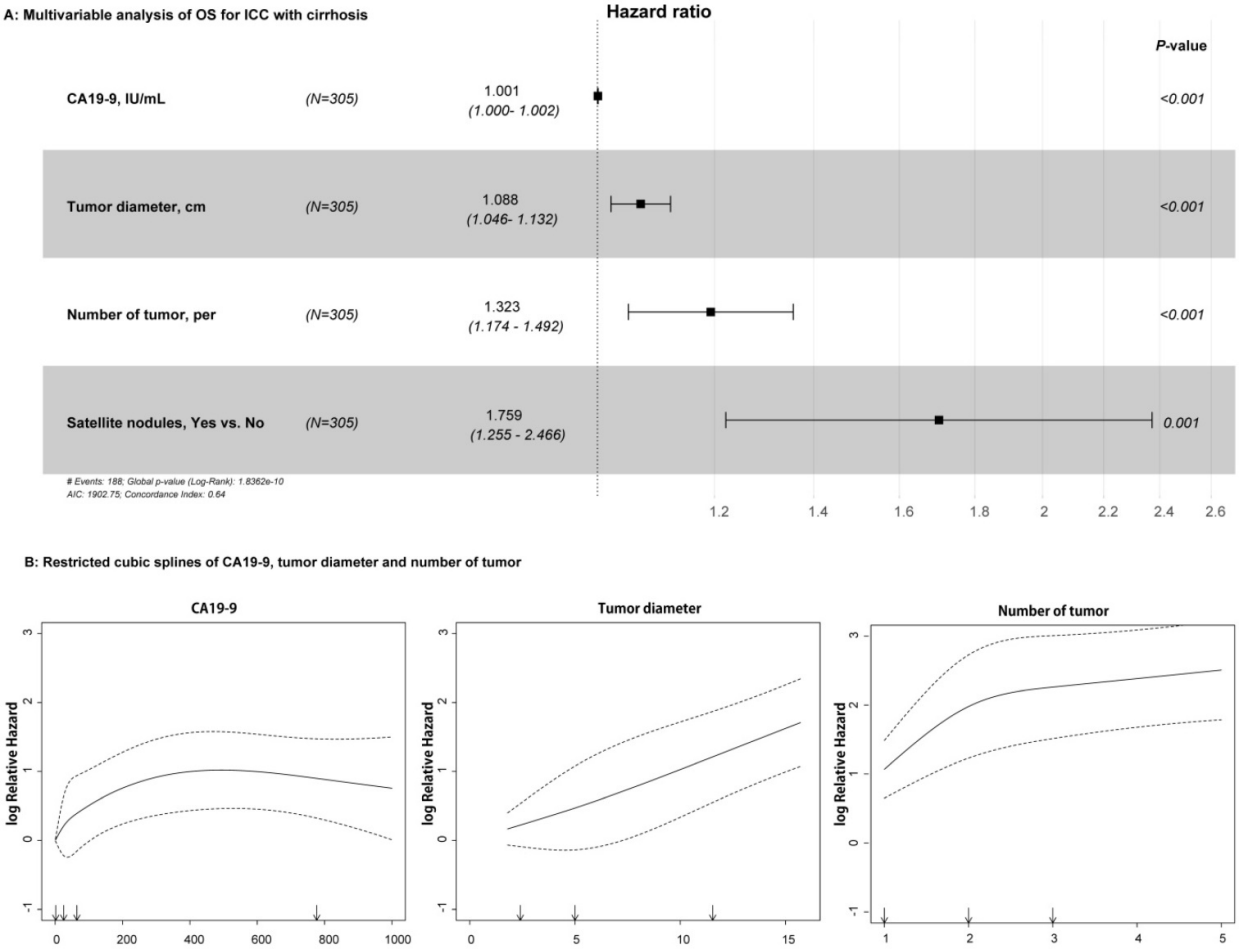
Relationships between the variables and OS rate of patients with ICC and cirrhosis. **A:** Forest plots summarizing the multivariable result of OS; **B:** Nonlinear relationships of CA19-9 value, tumor diameter, and number with OS of patients with ICC and cirrhosis.

For the univariable analysis, presence of CSPH (HR=1.399, 95%CI=1.038-1.886, *p* = 0.027), high CA19-9 value (HR=1.001, 95%CI=1.000-1.002, *p* < 0.001), large tumor diameter (HR=1.118, 95%CI=1.078-1.159, *p* < 0.001), more tumor numbers (HR=1.437, 95%CI=1.287-1.604, *p* < 0.001), and presence of satellite nodules (HR=1.747, 95%CI=1.257-2.429, *p* = 0.001) were related to OS in the training cohort (Table 2). After multivariable Cox regression analysis, high CA19-9 value (HR=1.001, 95%CI=1.000-1.002, *p* < 0.001), large tumor diameter (HR=1.088, 95%CI=1.046-1.132, *p* < 0.001), more tumor numbers (HR=1.323, 95%CI=1.174-1.492, *p* < 0.001), and satellite nodules (HR=1.759, 95%CI=1.255-2.466, *p* = 0.001) were the independent risk factors of OS (Table 2 and Figure 2A). As shown in Figure 2B, RCS showed that nonlinear relationships existed between the factors of CA19-9, radiological tumor diameter, and tumor number and OS rate in ICC patients with cirrhosis. As the tumor diameter and number increased, the patient's risk of death increased. However, when CA19-9 > 500 IU/mL, the risk of death decreased slightly. In addition, the results of RFS in univariable and multivariable Cox analyses are shown in Table S1.

### Establishment of the EHBH model

**Figure 3:**
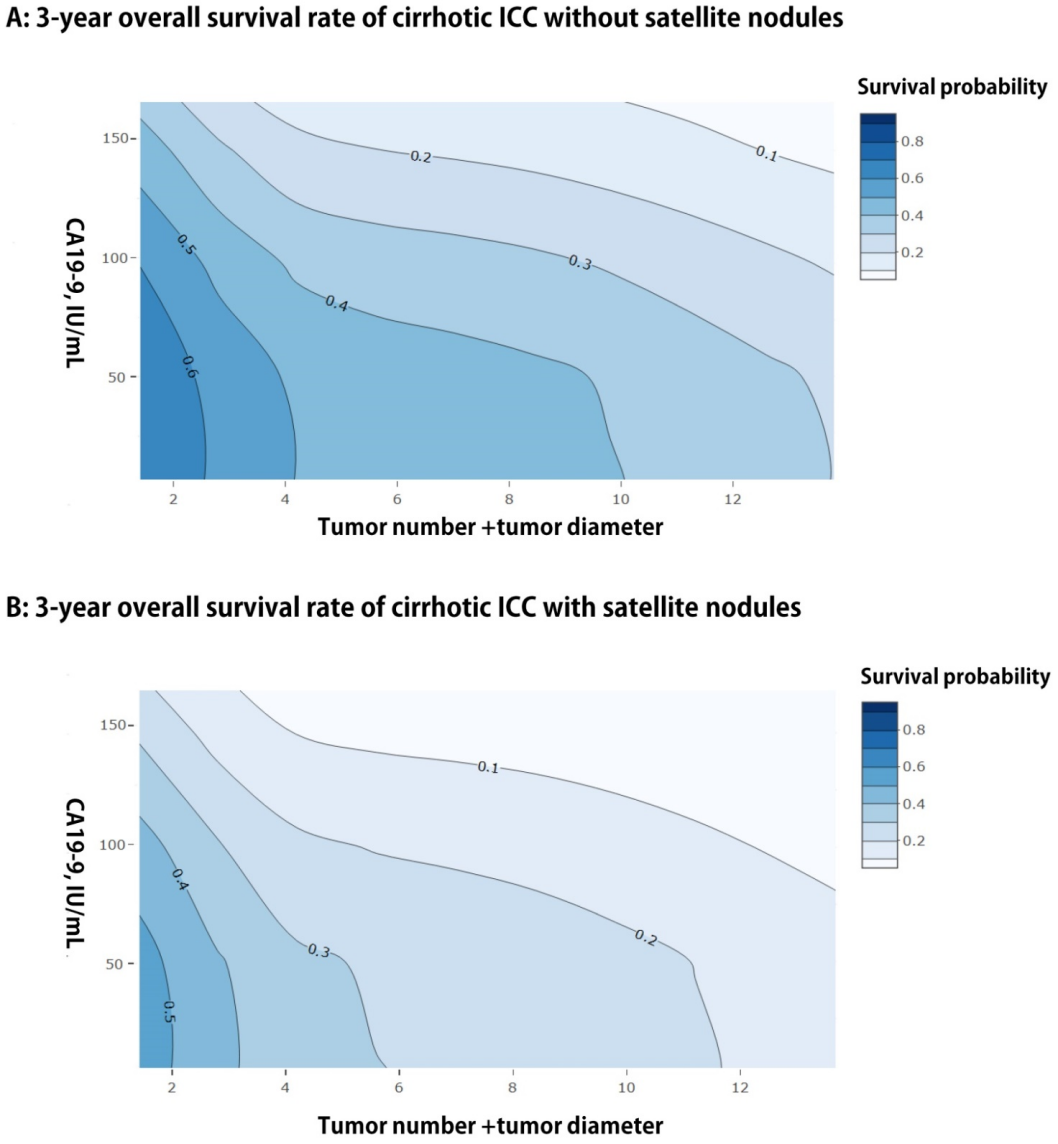
Contour plots of three-year using the EHBH model for ICC patients with cirrhosis. **A:** For patients without satellite nodules. **B:** For patients with satellite nodules.

We presented a simple EHBH model consisted of a serological index and three preoperative imaging features, namely, CA19-9, tumor diameter, tumor number, and satellite nodules, based on the result of multivariable analysis for OS in the training cohort. The EHBH model can be used to visually predict the overall 3-year survival probabilities of all patients with cirrhosis, as shown in Figures 3A and 3B with two contour plots. The contour plot included two steps. First, the specific contour plot should be selected for the patients whether with or without satellite nodules (without satellite nodules = A, with satellite nodules = B). Second, the predicted probability was traced by tumor number + tumor diameter (X-axis) and CA19-9 value (Y-axis) in the specific contour plot. Moreover, the one- and five-year predicted survival probabilities are shown in Figure S2, which used the same way as previously described.

**Figure 4:**
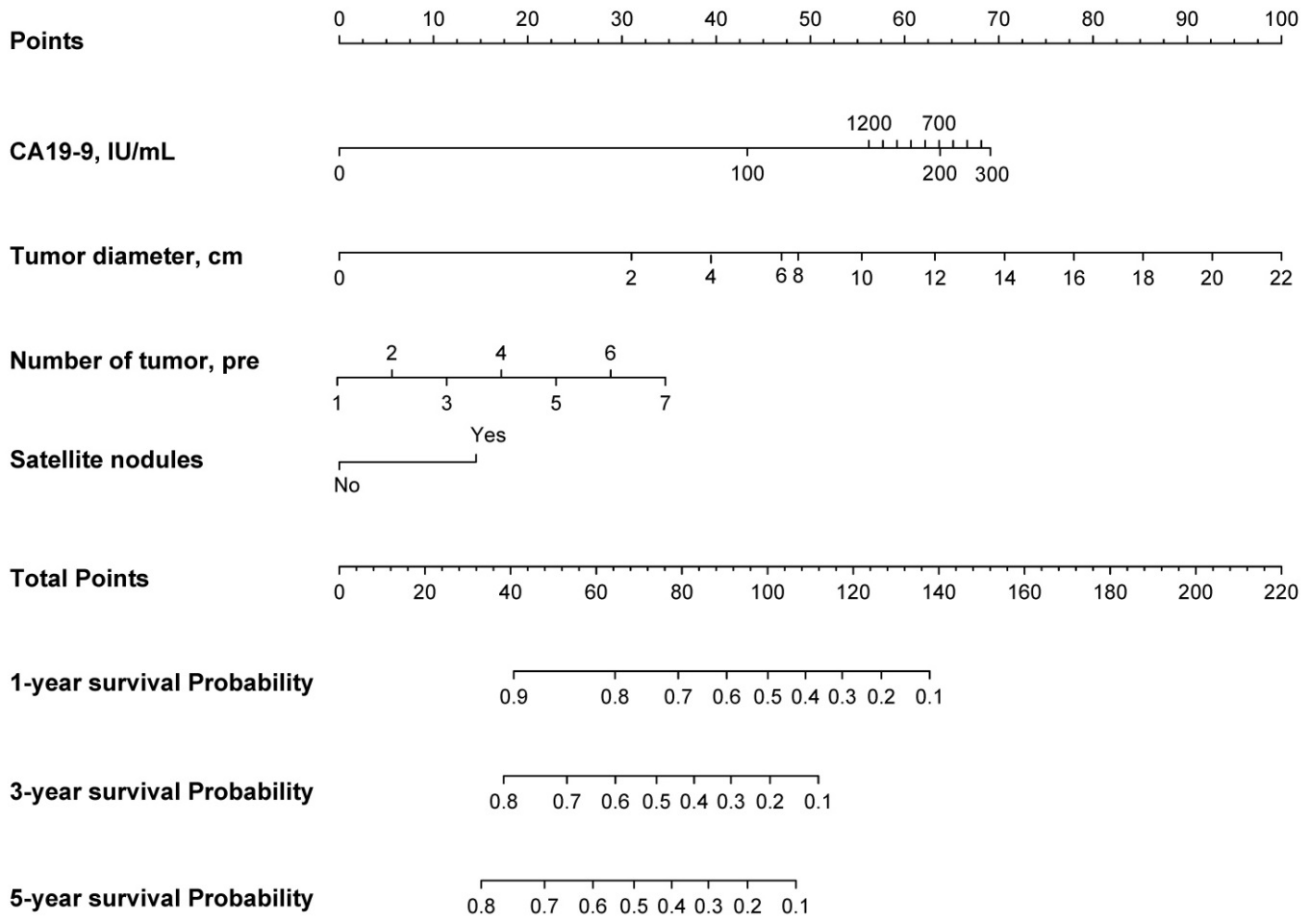
Nomogram of the EHBH model for individual survival prediction.

Based on the EHBH model, a nomogram was built for individual patient survival risk stratification (Figure 4). The 1-year, 3-year, and 5-year survival probabilities of individual patients could be predicted before the surgery with the sum of CA19-9 value, tumor size, tumor number, and satellite nodules.

### Risk stratification and selection for optimal surgical patients

To indicate the efficacy of the EHBH model to select reasonably ICC patients with cirrhosis for hepatectomy, we calculated the nomogram score for each patient in the training cohort and through ROC curves found the best cut-off value of 86.56 points, and then divided the patients into high-risk and low-risk groups, as shown in Figure S3.

**Figure 5:**
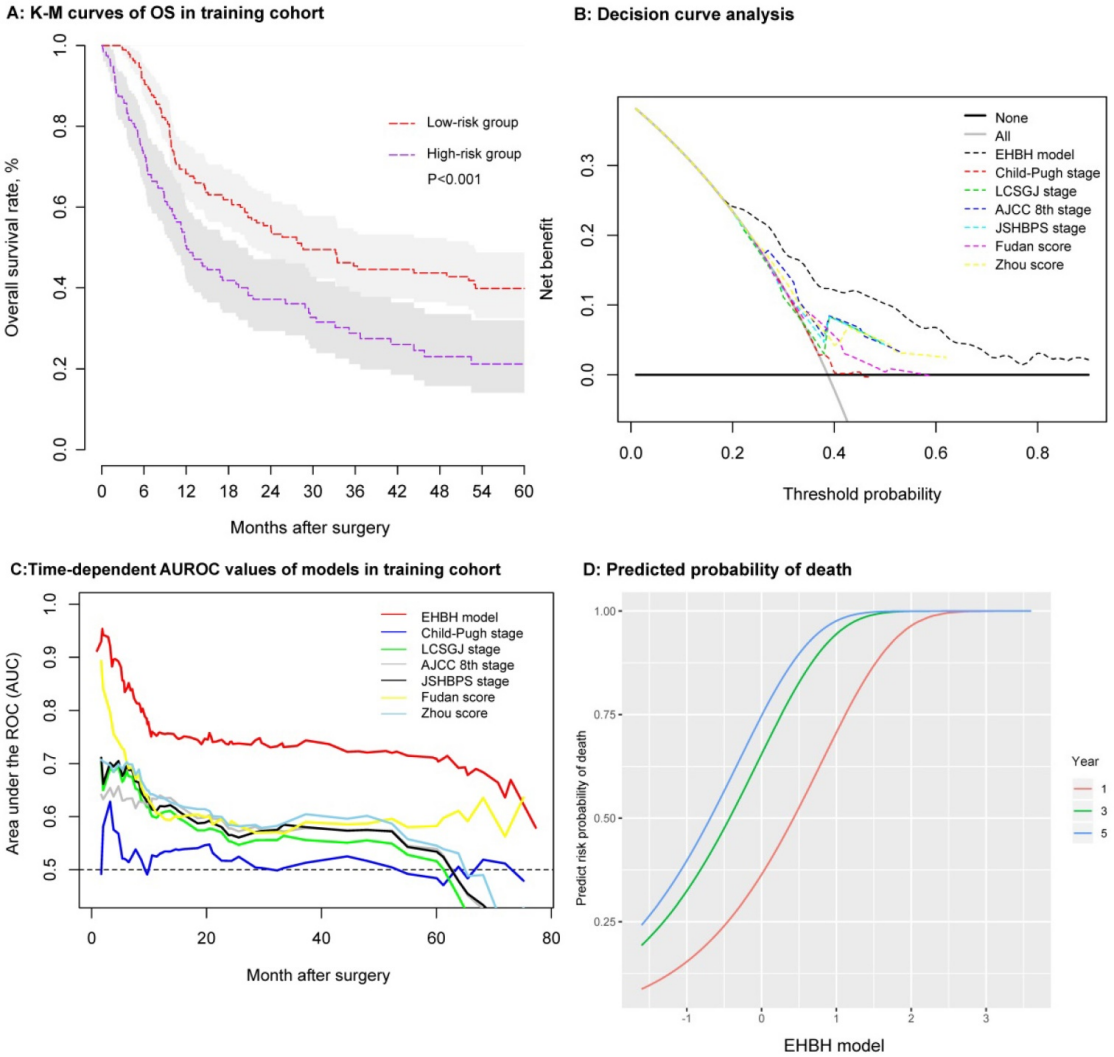
Establishment and assessment of the EHBH model in the training cohort. **A:** Kaplan-Meier curve of the stratified EHBH model in the training cohort. **B:** DCA of the EHBH model. **C:** Time-dependent ROC for the EHBH model and other clinical staging systems in the training cohort. **D:** Predicted probability of one-, three-, and five-year death using the EHBH model.

As shown in Figure 5A, the patients' survival outcomes of the high-risk group (n = 119) were significantly worse than those of the low-risk group (n = 186) (median time, 11.91 vs. 28.39 months, p < 0.001) in the training cohort. The one-, three-, and five-year OS rates were 50.4%, 29.0%, and 21.0% for the high-risk group and 68.2%, 45.5%, and 39.7% for the low-risk group, respectively. The one-, three-, and five-year RFS rates using the same cut-off value of 86.56 in the training cohorts were 38.9%, 28.7%, and 20.2% for the high-risk group and 55.6.9%, 35.2%, and 32.5% for the low-risk group, respectively (p = 0.004), as shown in Figure S1C.

### Assessment and comparison of the EHBH model

Several methods were used to verify the predictive accuracy of the EHBH model. First, DCA was used to facilitate the comparison between the EHBH model and other staging systems (Child-Pugh stage, LCSGJ stage, AJCC 8th stage, JSHBPS stage, Fudan score, and Zhou score) in the training cohort. As shown in Figure 5B, DCA demonstrated that the EHBH-HVTT scoring system provided superior net benefit when the threshold value >0.2 compared with other commonly used international staging systems. Table S2 shows that the EHBH model with the highest C-index (C-index = 0.710, 95%CI = 0.673-0.748) was significantly better than the common staging systems under Harrell's c-statistics (all p < 0.0001). Furthermore, time-dependent-ROC curve area analysis was used to determine which staging systems were good at predicting the survival outcomes. As shown in Figure 5C, the EHBH model outperformed the other six currently available models in the training cohort. The time-dependent AUC ranging from 10 months to 80 months for the EHBH model was 0.68 (0.59-0.78) in the training cohort.

These results demonstrated that the EHBH model had a higher diagnostic capacity and better AUC than the other six staging systems in cirrhotic patients with ICC. The predicted plots of OS probabilities (one-, three-, and five-year) in each clinical staging system for all cirrhotic ICC patients are shown in Figure 5D and Figure S4. The EHBH model showed a significant relationship with the largest Wald and Slope with the OS rate of patients compared with other staging systems.

### Validation of the EHBH model

**Figure 6:**
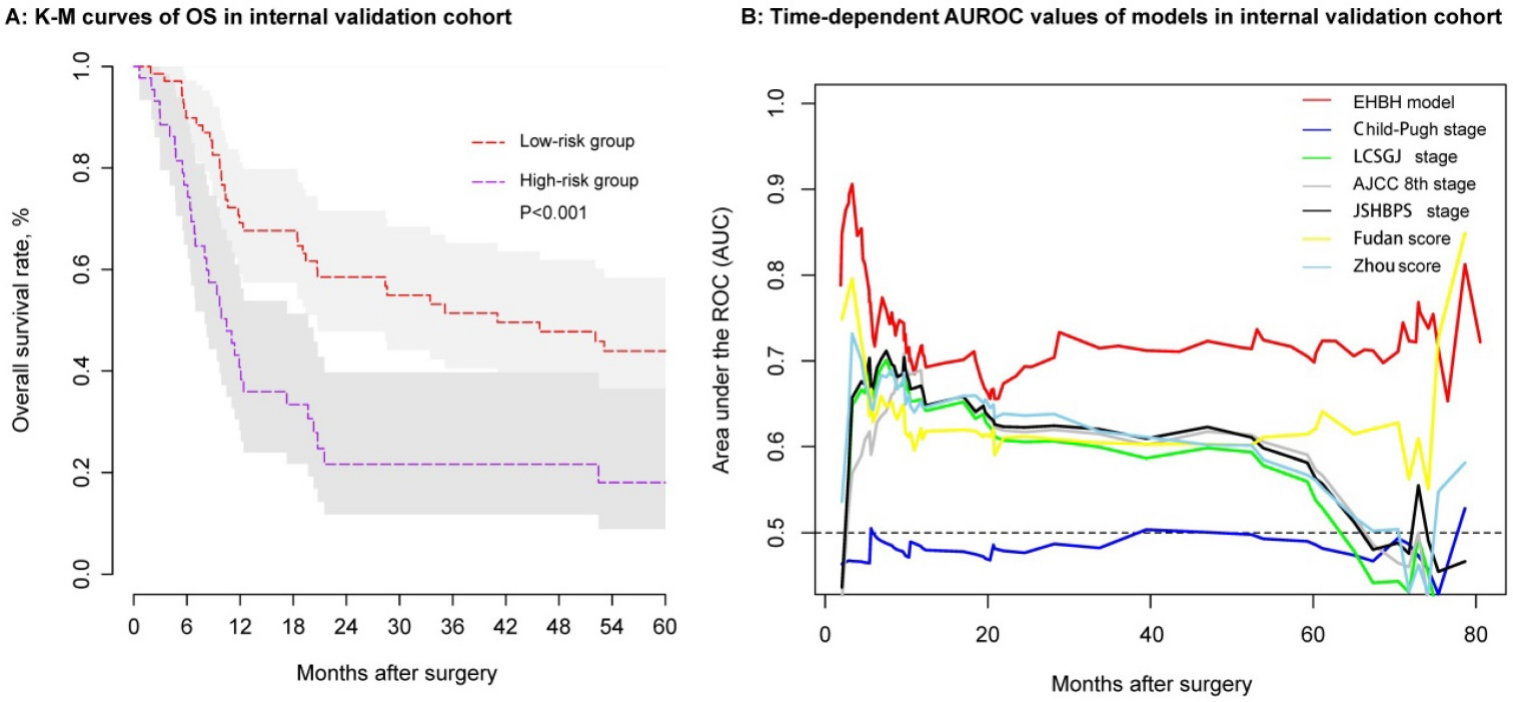
Assessment of the EHBH model in the internal validation cohort. **A:** Kaplan-Meier curve of the stratified EHBH model in the internal validation cohort. **B:** Time-dependent ROC for the EHBH model and other clinical staging systems in the internal validation cohort.

Using the same threshold of 86.56 points, the identification ability of the internal validation cohort performed equally well with the EHBH model established in the training cohort. In the internal validation cohort, ICC patients in the low-risk group (n = 69) had a longer median OS than that in the high-risk group (n = 44) (Figure 6A, median time, 41.05 vs. 10.47 months, p < 0.001). The EHBH model can easily distinguish the optimal patients with better RFS (p=0.040) (Figure S1D). Moreover, time-dependent AUC ranging from 10 months to 80 months of the EHBH model in the internal validation cohort was 0.72 (0.64-0.82) (Figure 6B). As shown in Figure 6B, the AUC value of the EHBH model was the highest in all staging systems at a time range from 10 months to 73 months.

## Discussion

Cirrhosis is regarded as an important negative prognostic factor for the survival of ICC patients. The incidence of cirrhosis has been estimated to be 27.8% to 50.5% in ICC patients 21, 22. With the advancement of surgical techniques 22-27, more and more cirrhotic patients are suitable for surgery. However, survival outcomes are still controversial, especially in patients with multiple tumors 28.

To the best of our knowledge, this study was the first to establish a simple model using preoperative indexes for the decision making on hepatectomy and prognosis prediction of ICC patients with cirrhosis, especially for those with multiple tumors. A few of previous studies have established preoperative prognosis models for ICC, however, some studies just use single preoperative indicators or indexes to predict the prognosis of ICC, such as albumin-bilirubin grade (ALBI), albumin-to-alkaline phosphatase ratio (AAPR) 29, and preoperative prognostic nutritional index (PNI) 30. Those indexes did not contain any information about tumor characteristics, such as tumor size, number, and so on. Some studies use multivariable regression to select significant factors and then develop a risk score to accomplish survival prediction 31, 32. Compared with ours, those risk score models require complex calculation process in clinical application. In the contrary, our EHBH model just consists of three factors, and the contour plots are easily used to estimate the survival probability of ICC patients. What' more, a nomogram can be used to distinguish the high-risk cirrhotic patients before surgery. The patients in the low-risk group (nomogram score ≤ 86.56 points) were good candidates for LR because they would have good median OS and RFS. A contour plot was used as a visualization tool for surgeons and patients to accomplish individualized prognosis prediction of 3-year survival probability based on preoperative CA19-9 value, tumor number + tumor diameter, and satellite nodules. Most importantly, the EHBH model performed better in the prognosis prediction than the other commonly used staging systems.

Cumulative evidences have proved CA19-9 useful to help surgeons selecting proper patients with intrahepatic cholangiocarcinoma (ICC) for surgery and an important preoperative biomarker for prognostic prediction after surgical treatment. For example, Yamamoto et al. 33 suggested that therapy or resection should be carefully determined in ICC patients with CA19-9 > 300 U/ml. Moreover, a study from He et al. 34 showed that ICC patients with preoperative CA19-9 value > 200 U/ml generally had a poor surgical result. In this study, our conclusion was consistent with the previous. A higher level of preoperative CA19-9 value was significantly associated with worse prognosis after LR in cirrhotic ICC patients.

Tumor size and number were also considered as important risk factors of ICC based on Wang's nomogram 3. Recently, a multicenter study demonstrated the linear effect of tumor size on survival 8. These findings supported the well-established relationship between tumor size and prognosis of HCC 35. Controversies have existed about the relationship between tumor number and survival in ICC after surgery. Some authors have identified multiple tumors as a negative prognostic factor 6-11. For example, Pietro et al. 36 indicated that multiple tumors are common in ICC patients who are characterized by a decrease in OS and DFS and a trend toward early recurrence. Other researchers have not considered that tumor number is a risk factor for survival 12.

Some researchers have pointed out that tumor multinodularity is associated with other poor prognostic factors in ICC 37. Pietro et al. 36 also observed that tumor multinodularity is associated with the increase in LNM rate, perineural, and vascular invasion, leading to poor prognosis of patients after LR. What's more, tumor multinodularity was associated with the highest rate of LNMs (62%), confirming similar findings of two previous studies 9, 12. In this study, we observed that tumor diameter and tumor number reflected the poor prognosis of ICC on multivariable analyses.

Recently, many studies have shown that satellite nodules are an important risk predictor of survival in patients with ICC 36, 38-40. Our results showed that satellite nodules could increase the death rate in ICC patients which are consistent with the recent study by Lu et al. 38. The research of Zhang et al. 40 also suggested that satellite lesions were an independent risk factor for recurrence in ICC.

Satellite nodules might be a presence of vascular infiltration suggesting a more aggressive form in ICCs, which could explain why patients with satellite nodules received worse survival outcomes in our study. Besides, satellite nodules usually combined with a higher rate of multiple tumors in ICC patients 36, 38. In the present study, we also found that the percentage of patients having > 2 nodules with satellite nodules was more than that without satellite nodules.

This study compared six common international staging systems to stratify ICC patients into different risk categories using the EHBH model. We found that those six staging systems had poor prediction capability of OS. This condition was because some systems were originally developed from mixed populations, and only part of patients involved in the establishment of those systems was suitable for surgery. On the other hand, the establishment of those systems just based on the clinicopathological data from hundreds of ICC patients.

The Child-Pugh system was originally developed to assess the prognosis of patients with cirrhosis and portal hypertension who were assessed to undergo surgery for variceal bleeding. Some of the variables included in this system were interrelated (e.g., ascites and serum albumin levels). Furthermore, the grading of ascites and encephalopathy was subjective 41.

The other five clinical staging systems, namely, LCSGJ stage, AJCC 8th stage, JSHBPS stage, Fudan score, and Zhou score based on tumor characteristics and blood biochemical had some limitations such as lacking external validations, including patients who were unsuitable for surgery and so on. Besides, these staging systems had limitations because they cannot be used to make an individualized prediction of survival probability before LR for ICC patients with cirrhosis. Our EHBH model can solve those problems using the contour plot of predicted 3-year survival probability based on the result of multivariable Cox regression in ICC before surgery. This model can identify cirrhotic ICC patients with proper tumor number and size suitable for surgery.

The EHBH model has four significant advantages in clinical practice compared with other staging systems for ICC patients with cirrhosis. First, the model is simple and only contains four variables. Second, the EHBH model aids surgeons to select suitable patients for LR. Third, the contour plot can achieve individualized prediction for long-term survival. Lastly, this new system is stable and accurate in predicting the OS of cirrhotic ICC patients. Taken together, the EHBH model can be used as a supplement to the commonly used international staging systems to improve their prognostic ability in cirrhotic ICC patients.

The current study had several limitations. First, it only involved one single institution in China, and patients were all cirrhotic background. The model should be validated using more external cohorts with different backgrounds. Second, the EHBH model using preoperative radiological indexes need to further improve the accuracy of prognosis prediction with more preoperative parameters. Third, this retrospective study was inevitably exposed to selection bias, and other potential confounding factors that may have prognostic roles were not adjusted.

All in all, the EHBH model was used as a selection aid for surgeons to screen cirrhotic ICC patients suitable for hepatectomy and predict the individual survival probability of ICC patients with cirrhosis, which can be used as a supplement to other ICC staging systems.

## Supplementary Material

Supplementary figures and tables.

## Figures and Tables

**Table 1:** Baseline characteristics of the study cohorts

Variable	Entire cohort (n=418)	Training cohort (n=305)	Validation cohort (n=113)	*P* value
**Basical results**				
Age, Year	51.8 ± 10.3	52.1 ± 10.2	51.2 ± 10.5	0.455
**Gender, n (%)**				0.494
Male	368.0 (88.0)	266.0 (87.2)	102.0 (90.3)	
Female	50.0 (12.0)	39.0 (12.8)	11.0 (9.7)	
BMI	24.2 ± 4.0	24.2 ± 3.9	24.1 ± 4.0	0.814
**CSPH, n (%)**				0.464
Yes	131.0 (31.3)	92.0 (30.2)	39.0 (34.5)	
No	287.0 (68.7)	213.0 (69.8)	74.0 (65.5)	
**Diabetes, n (%)**				0.775
Yes	29.0 (6.9)	20.0 (6.6)	9.0 (8.0)	
No	389.0 (93.1)	285.0 (93.4)	104.0 (92.0)	
**HBsAg, n (%)**				0.545
Positive	365.0 (87.3)	264.0 (86.6)	101.0 (89.4)	
Negative	53.0 (12.7)	41.0 (13.4)	12.0 (10.6)	
**HCV-Ab, n (%)**				1.000
Positive	10.0 (2.4)	7.0 (2.3)	3.0 (2.7)	
Negative	408.0 (97.6)	298.0 (97.7)	110.0 (97.3)	
**Serological results**				
TBIL, mg/dL*	0.8 (0.6, 1.0)	0.8 (0.6, 1.0)	0.7 (0.7, 1.1)	0.366
ALB, g/L	41.3 ± 4.9	41.2 ± 4.8	41.5 ± 5.0	0.574
ALT, IU/L*	35.5 (25.6, 56.0)	35.3 (25.5, 55.6)	36.4 (25.7, 58.0)	0.636
AST, IU/L*	32.1 (25.0, 45.6)	31.9 (25.0, 44.2)	32.7 (25.0, 51.1)	0.261
GGT, IU/L*	67.5 (40.0, 149.0)	67.0 (41.0, 148.5)	75.0 (38.0, 151.0)	0.922
PT, second *	12.2 (11.7, 13.0)	12.2 (11.7, 13.0)	12.2 (11.7, 13.1)	0.642
PLT, ×10^9^/L	151.45 ± 64.39	152.54 ± 65.28	148.51 ± 62.13	0.571
AFP, μg/L*	8.6 (3.7, 65.2)	8.4 (3.6, 56.1)	9.8 (4.0, 112.5)	0.296
CEA, μg/L*	2.6 (1.7, 4.2)	2.5 (1.6, 4.1)	2.7 (1.7, 4.7)	0.452
CA19-9, IU/mL*	39.5 (17.9, 121.0)	39.7 (18.1, 124.3)	36.4 (17.3, 92.7)	0.503
**Child-Pugh grade, n (%)**				0.781
A	384.0 (91.9)	279.0 (91.5)	105.0 (92.9)	
B	34.0 (8.1)	26.0 (8.5)	8.0 (7.1)	
**Imaging results**				
Tumor diameter, cm*†	5.0 (3.5, 8.0)	5.0 (3.6, 8.0)	4.9 (3.2, 8.5)	0.995
**Tumor distribution, n (%)**				0.887
Bilateral	59.0 (14.1)	44.0 (14.4)	15.0 (13.3)	
Unilateral	359.0 (85.9)	261.0 (85.6)	98.0 (86.7)	
**Number of tumor nodal**				0.967
Multiple	169.0 (40.4)	124.0 (40.7)	45.0 (39.8)	
Sigle	249.0 (59.6)	181.0 (59.3)	68.0 (60.2)	
**Satellite nodules, n (%)**				0.048
Yes	96.0 (23.0)	62.0 (20.3)	34.0 (30.1)	
No	322 (77.0)	243 (79.7)	79 (69.9)	
**Tumor capsule, n (%)**				0.718
No	342 (81.8)	252 (82.6)	90 (79.6)	
Complete	37 (8.9)	25 (8.2)	12 (10.6)	
Incomplete	39 (9.3)	28 (9.2)	11 (9.7)	

**Abbreviations:** CSPH, clinically significant portal hypertension; BMI, Body Mass Index; HBsAg, hepatitis B surface antigen; HCV, hepatitis C virus; TBIL, total bilirubin; ALB, albumin; ALT, alanine transaminase; AST, aspartate aminotransferase; GGT, gamma-glutamyl transpeptidase; PT, prothrombin time; PLT, platelet; AFP, alpha-fetoprotein; CEA, carcinoembryonic antigen; CA19-9, carbonhydrateantigen19-9. † Largest tumor diameter in cm. * Quantitative variables are shown with median and interquartile range (IQR).

**Table 2:** Univariable and multivariable analyses of overall survival

Variable	Univariable analysis			Multivariable analysis		
	HR	95%CI	*P* value	HR	95%CI	*P* value
Age, year	1.001	0.988 - 1.015	0.846			
Gender, Male vs. Female	1.327	0.842 - 2.090	0.221			
BMI	0.977	0.942 - 1.014	0.223			
CSPH, Yes vs. No	1.399	1.038 - 1.886	0.027			
Diabetes, Yes vs. No	1.363	0.790 - 2.352	0.263			
HBsAg, Yes vs. No	0.966	0.643 - 1.453	0.870			
Anti-HCV, Yes vs. No	0.517	0.165 - 1.619	0.249			
TBIL, mg/dL	1.019	0.964 - 1.076	0.504			
ALB, g/L	0.987	0.958 - 1.018	0.423			
ALT, IU/L	1.000	0.999 - 1.001	0.416			
AST, IU/L	1.000	0.999 - 1.001	0.263			
GGT, IU/L	1.000	0.999 - 1.001	0.920			
PT, second	1.071	0.953 - 1.203	0.250			
PLT, ×10^9^/L	1.000	0.998 - 1.003	0.729			
AFP, μg/L	1.000	0.999 - 1.001	0.759			
CEA, μg/L	1.001	0.999 - 1.003	0.101			
CA19-9, IU/mL	1.001	1.000 - 1.002	<0.001	1.001	1.000-1.002	<0.001
Child-Pugh grade, B vs. A	1.164	0.716 - 1.893	0.539			
Tumor diameter, cm	1.118	1.078 - 1.159	<0.001	1.088	1.046-1.132	<0.001
Tumor distribution, Bilateral vs. Unilateral	1.212	0.825 - 1.783	0.326			
No. of tumor, per	1.437	1.287 - 1.604	<0.001	1.323	1.174-1.492	<0.001
Satellite nodules, Yes vs. No	1.747	1.257 - 2.429	0.001	1.759	1.255-2.466	0.001
Tumor capsule, Yes vs. No	0.843	0.652 - 1.089	0.189			

**Abbreviations:** CSPH, clinically significant portal hypertension; BMI, Body Mass Index; HBsAg, hepatitis B surface antigen; HCV, hepatitis C virus; TBIL, total bilirubin; ALB, albumin; ALT, alanine transaminase; AST, aspartate aminotransferase; GGT, gamma-glutamyl transpeptidase; PT, prothrombin time; PLT, platelet; AFP, alpha-fetoprotein; CEA, carcinoembryonic antigen; CA19-9, carbonhydrateantigen19-9.
