# Supplementary material for: Developing a Selection-aided Model to Screen Cirrhotic Intrahepatic Cholangiocarcinoma for Hepatectomy
Source: J Cancer. 2020 Jul 25;11(19):5623–34. doi: 10.7150/jca.46587 (PMC7477447; doi:10.7150/jca.46587)
Supplement: Supplementary file 1 — Supplementary figures and tables. [file jcav11p5623s1.pdf]

Content of supplementary files:

Figure S1. A and B: Comparison of Kaplan–Meier curves in OS and RFS between the two cohorts. C: Kaplan–Meier curve of the stratified EHBH model in the training cohort D: Kaplan–Meier curve of the stratified EHBH model in the internal validation cohort.

Figure S2. Contour plot of one- and five-year survival probabilities.

Figure S3. ROC curves of nomogram score in predicting the death of training cohort.

Figure S4. Predicted probability of death in different models.

Table S1. Univariable and multivariable analyses of RFS.

Table S2. Comparison of the EHBH model and other clinical staging systems.

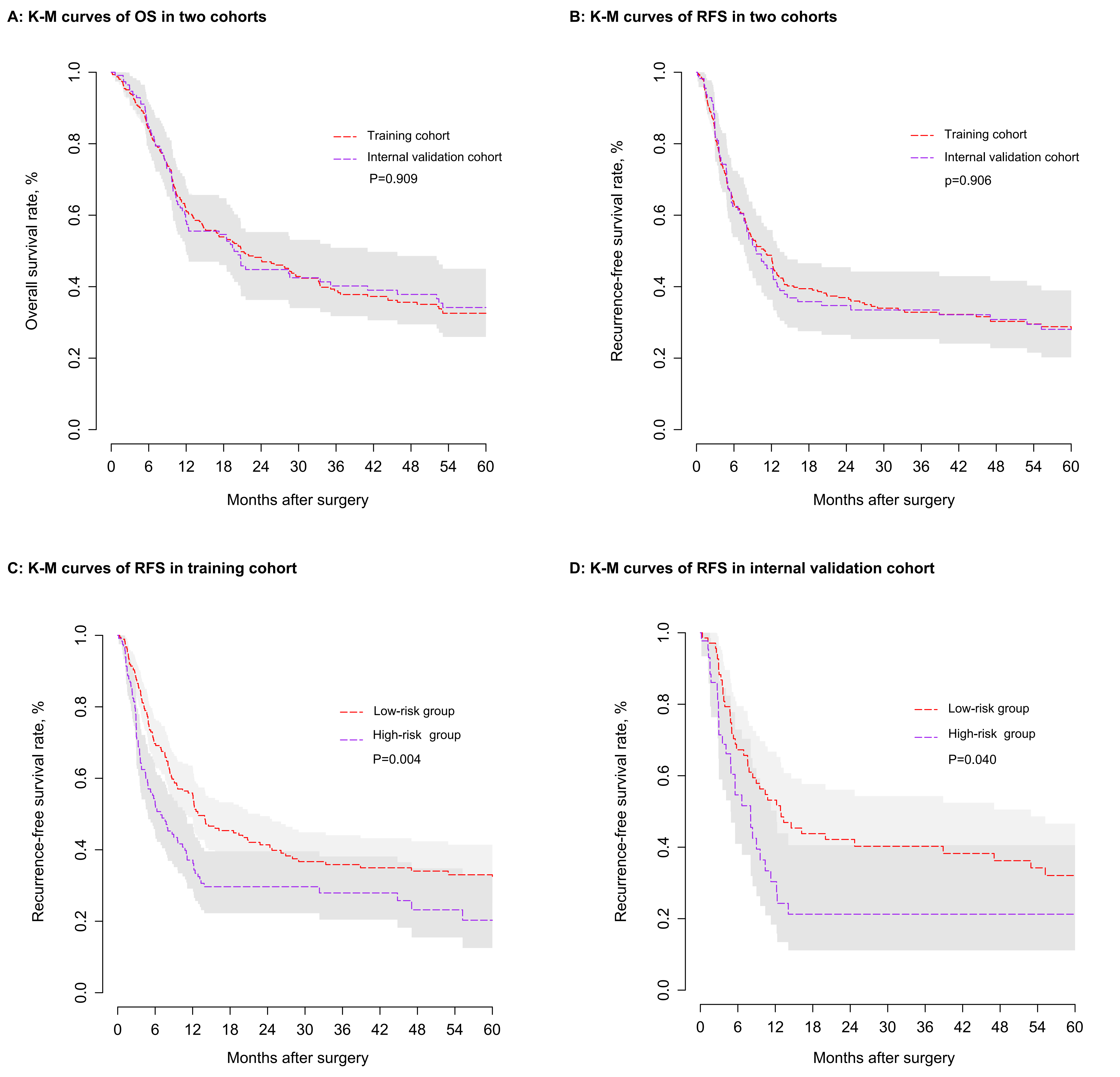

A: K-M curves of OS in two cohorts
Overall survival rate, %
1.0
0.8
0.6
0.4
0.2
0.0
Training cohort
Internal validation cohort
P=0.909
0
6
12
18
24
30
36
42
48
54
60
Months after surgery
B: K-M curves of RFS in two cohorts
Recurrence-free survival rate, %
Training cohort
Internal validation cohort
p=0.906
Months after surgery
C: K-M curves of RFS in training cohort
Recurrence-free survival rate, %
Low-risk group
High-risk group
P=0.004
Months after surgery
D: K-M curves of RFS in internal validation cohort
Recurrence-free survival rate, %
Low-risk group
High-risk group
P=0.040
Months after surgery

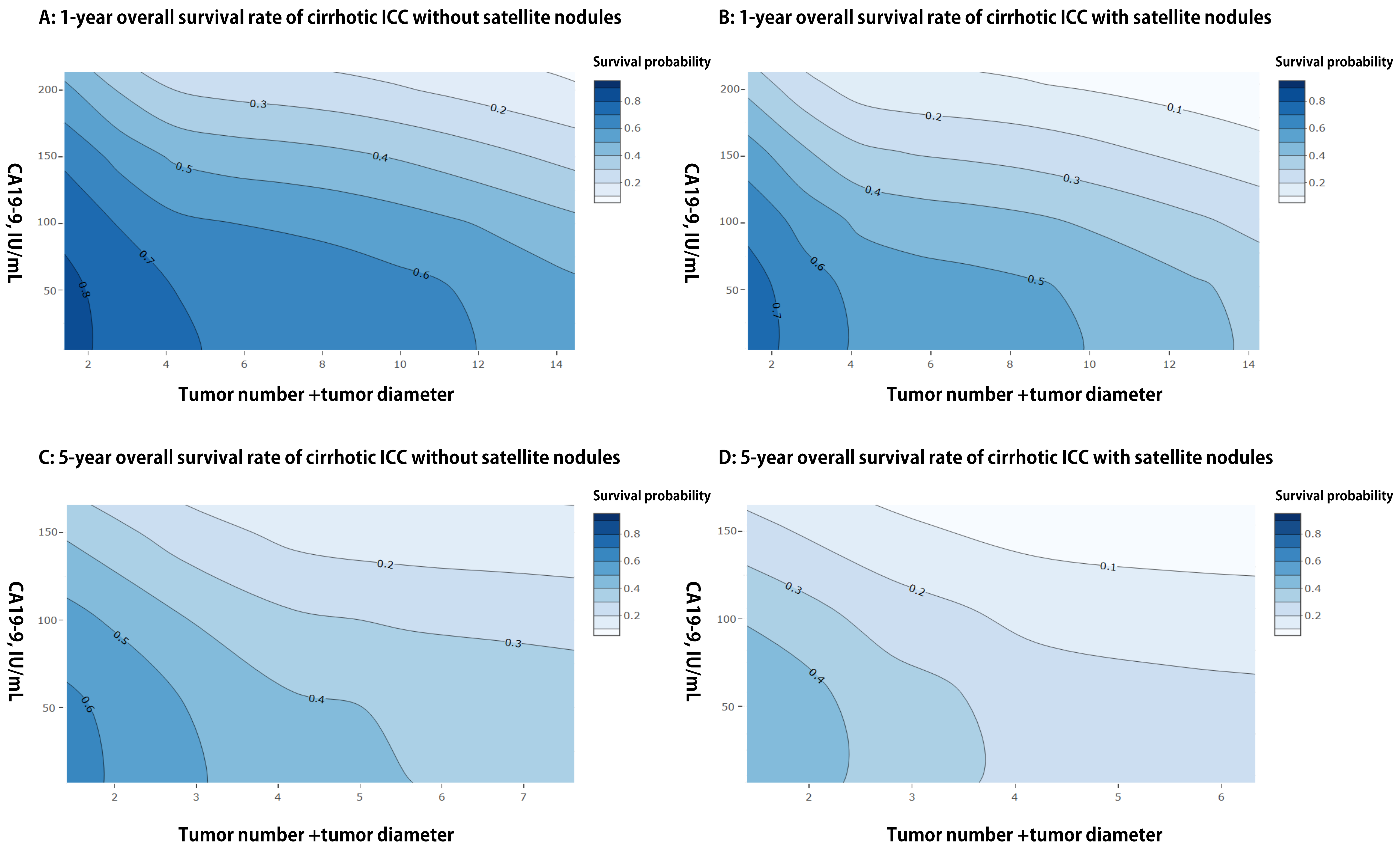
A: 1-year overall survival rate of cirrhotic ICC without satellite nodules
B: 1-year overall survival rate of cirrhotic ICC with satellite nodules
C: 5-year overall survival rate of cirrhotic ICC without satellite nodules
D: 5-year overall survival rate of cirrhotic ICC with satellite nodules
Survival probability
0.8
0.6
0.4
0.2
CA19-9, IU/mL
Tumor number +tumor diameter
200
150
100
50
2
4
6
8
10
12
14
0.8
0.7
0.6
0.5
0.4
0.3
0.2
0.1

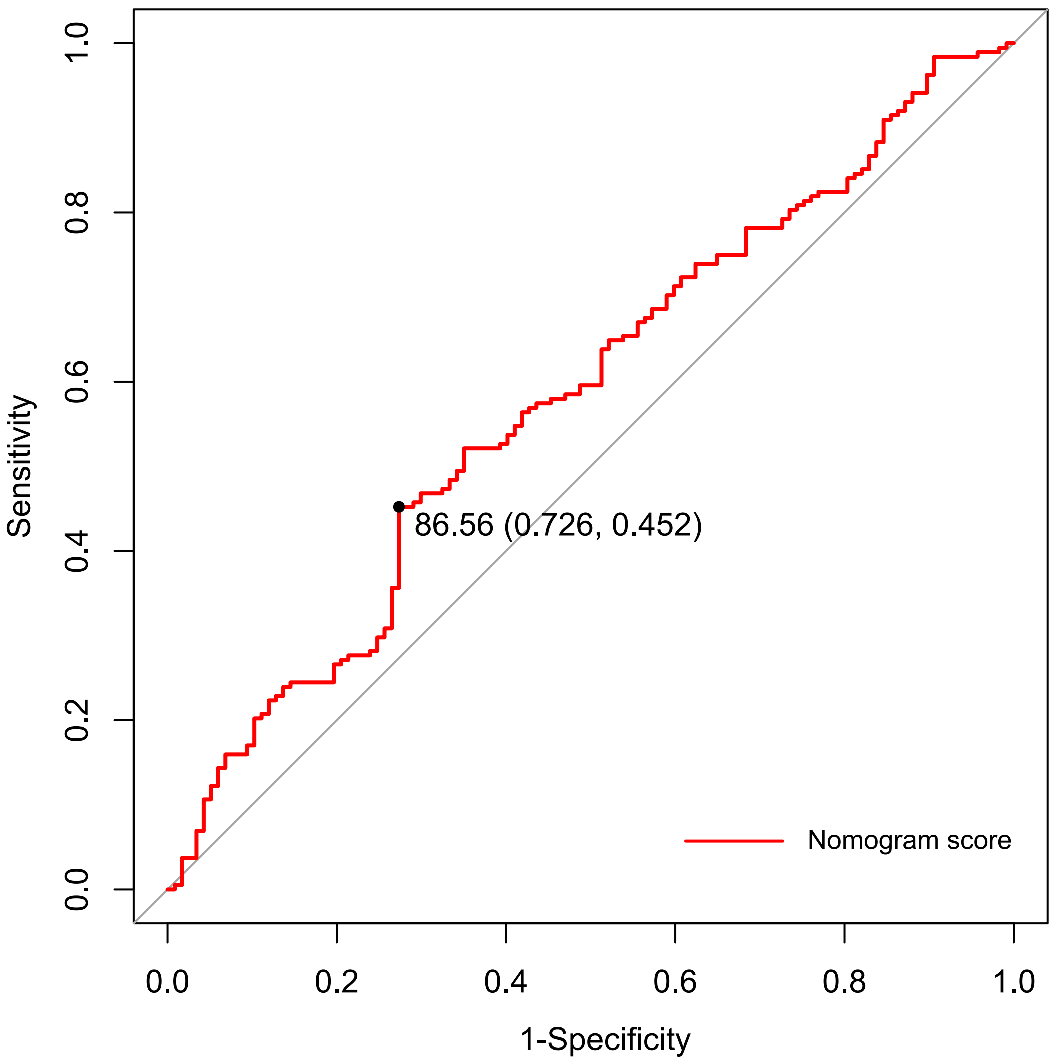

Sensitivity
1-Specificity
0.0
0.2
0.4
0.6
0.8
1.0
86.56 (0.726, 0.452)
Nomogram score

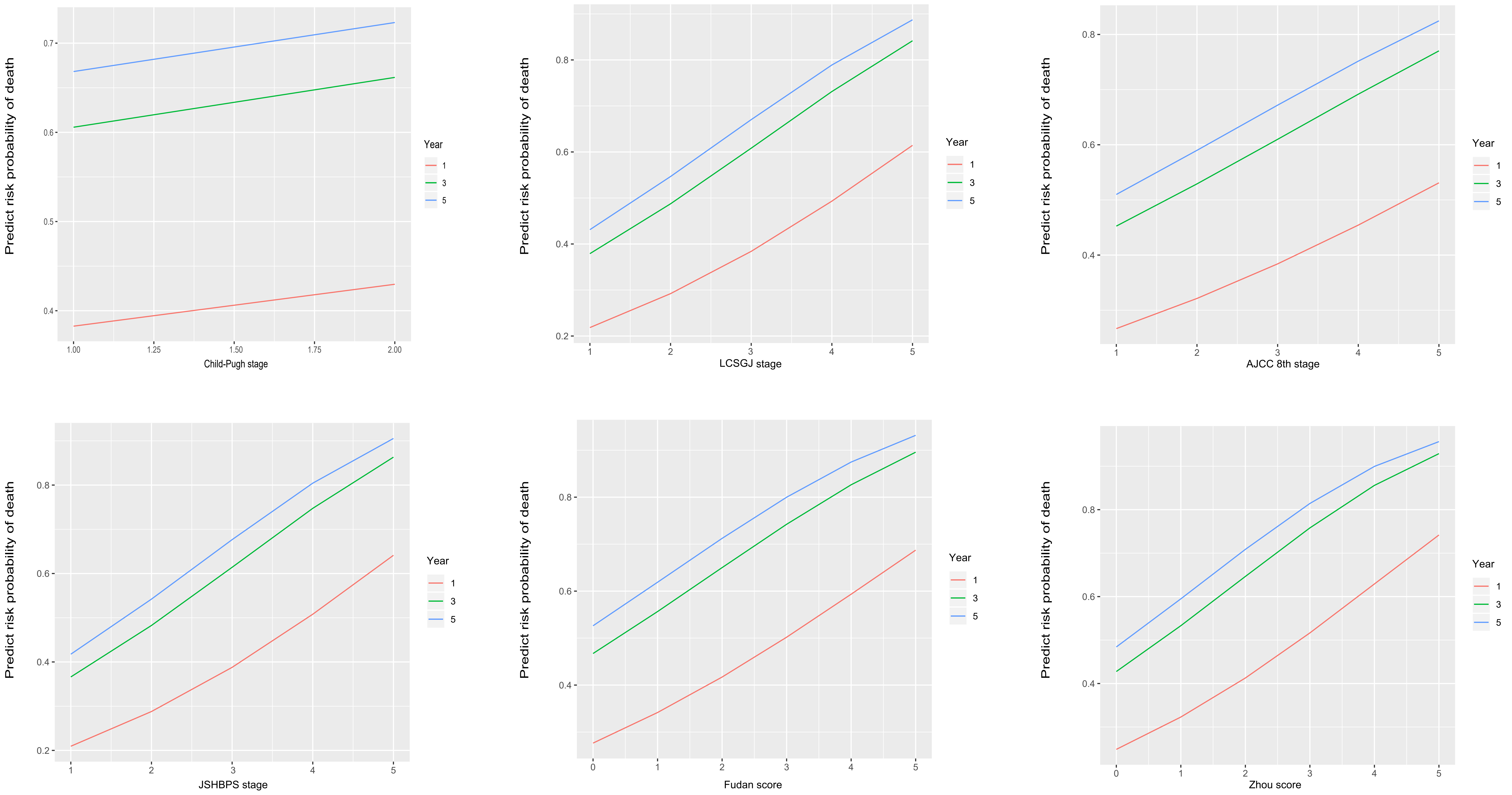
Predict risk probability of death
0.7
0.6
0.5
0.4
1.00
1.25
1.50
1.75
2.00
Child-Pugh stage
Year
1
3
5
Predict risk probability of death
0.8
0.6
0.4
0.2
1
2
3
4
5
LCSGJ stage
Year
1
3
5
Predict risk probability of death
0.8
0.6
0.4
1
2
3
4
5
AJCC 8th stage
Year
1
3
5
Predict risk probability of death
0.8
0.6
0.4
0.2
1
2
3
4
5
JSHBPS stage
Year
1
3
5
Predict risk probability of death
0.8
0.6
0.4
0
1
2
3
4
5
Fudan score
Year
1
3
5
Predict risk probability of death
0.8
0.6
0.4
0
1
2
3
4
5
Zhou score
Year
1
3
5

Table S1. Univariable and multivariable analyses of RFS

Variable	Univariable analysis			Multivariable analysis		
	HR	95%CI	*P* value	HR	95%CI	*P* value
Age, year	1.002	0.989 - 1.015	0.787			
Gender, Male vs. Female	1.253	0.817 - 1.921	0.301			
BMI	0.981	0.946 - 1.017	0.297			
CSPH, Yes vs. No	1.237	0.918 - 1.667	0.161			
Diabetes, Yes vs. No	1.275	0.740 - 2.196	0.380			
HBsAg, Yes vs. No	0.955	0.640 - 1.425	0.823			
Anti-HCV, Yes vs. No	0.301	0.075 - 1.215	0.074			
TBIL, mg/dL	0.981	0.916 - 1.052	0.593			
ALB, g/L	0.997	0.969 - 1.027	0.854			
ALT, IU/L	1.000	0.999 - 1.001	0.338			
AST, IU/L	1.000	0.999 - 1.001	0.161			
GGT, IU/L	1.000	0.999 - 1.001	0.904			
PT, second	1.070	0.943 - 1.214	0.293			
PLT, $\times 10^9$/L	1.000	0.998 - 1.002	0.919			
AFP, µg/L	1.000	0.999 - 1.001	0.592			
CEA, µg/L	1.003	1.001 - 1.005	0.001			
CA19-9, IU/mL	1.001	0.999 - 1.002	<0.001	1.001	1.000-1.002	0.001
Child-Pugh grade, B vs. A	0.787	0.457 - 1.356	0.388			

Total tumor diameter, cm	1.084	1.044 - 1.125	<0.001	1.055	1.013-1.104	0.010
Tumor distribution, Bilateral vs. Unilateral	1.096	0.743 - 1.617	0.643			
No. of tumor, per	1.478	1.311 - 1.667	<0.001	1.412	1.241-1.608	<0.001
Satellite nodules, Yes vs. No	1.579	1.134 - 2.197	0.006	1.607	1.151-2.246	0.005
Tumor capsule, Yes vs. No	0.980	0.784 - 1.225	0.862			

Abbreviations: CSPH, clinically significant portal hypertension; BMI, Body Mass Index; HBsAg, hepatitis B surface antigen; HCV, hepatitis C virus; TBIL, total bilirubin; ALB, albumin; ALT, alanine transaminase; AST, aspartate aminotransferase; GGT, gamma-glutamyl transpeptidase; PT, prothrombin time; PLT, platelet; AFP, alpha-fetoprotein; CEA, carcinoembryonic antigen; CA19-9, carbonhydrateantigen19-9.

Table S2. Comparison of the EHBH model and other clinical staging systems

	C-index (95%CI)	Change (95%CI)	*P* change
**EHBH model**	0.710 (0.673-0.748)		
**Child-Pugh stage**	0.519 (0.481-0.558)	-0.191 (-0.244 — -0.140)	**<0.0001**
**LCSGJ stage**	0.579 (0.537-0.620)	-0.131 (-0.170 — -0.093)	**<0.0001**
**AJCC 8th stage**	0.589 (0.551-0.628)	-0.121 (-0.159 — -0.084)	**<0.0001**
**JSHBPS stage**	0.589 (0.548-0.630)	-0.121 (-0.158 — -0.048)	**<0.0001**
**Fudan score**	0.594 (0.552-0.636)	-0.116 (-0.162 — -0.074)	**<0.0001**
**Zhou score**	0.598 (0.557-0.639)	-0.112 (-0.153 — -0.072)	**<0.0001**

Abbreviation: LCSGJ, liver cancer study group of Japan; AJCC: American Joint Committee on Cancer; JSHBPS, Japanese Society of Hepato-Biliary-Pancreatic Surgery.
